# Multi-source information fusion-driven corn yield prediction using the Random Forest from the perspective of Agricultural and Forestry Economic Management

**DOI:** 10.1038/s41598-024-54354-9

**Published:** 2024-02-19

**Authors:** Xuziqi Yang, Zekai Hua, Liang Li, Xingheng Huo, Ziqiang Zhao

**Affiliations:** 1 College of Economics and Management, Northwest A&F University, Yangling, 712100 Shaanxi China; 2 Laboratory of Walnut Research Center, College of Forestry, Northwest A&F University, Yangling, 712100 Shaanxi China; 3 College of Humanities and Social Development, Northwest A&F University, Yangling, 712100 Shaanxi China

**Keywords:** Agricultural and Forestry Economic Management, Multi source information fusion, Random Forest, Corn yield, Crop yield prediction model, Computational biology and bioinformatics, Environmental sciences, Mathematics and computing

## Abstract

The objective of this study is to promptly and accurately allocate resources, scientifically guide grain distribution, and enhance the precision of crop yield prediction (CYP), particularly for corn, along with ensuring application stability. The digital camera is selected to capture the digital image of a 60 m × 10 m experimental cornfield. Subsequently, the obtained data on corn yield and statistical growth serve as inputs for the multi-source information fusion (MSIF). The study proposes an MSIF-based CYP Random Forest model by amalgamating the fluctuating corn yield dataset. In relation to the spatial variability of the experimental cornfield, the fitting degree and prediction ability of the proposed MSIF-based CYP Random Forest are analyzed, with statistics collected from 1-hectare, 10-hectare, 20-hectare, 30-hectare, and 50-hectare experimental cornfields. Results indicate that the proposed MSIF-based CYP Random Forest model outperforms control models such as support vector machine (SVM) and Long Short-Term Memory (LSTM), achieving the highest prediction accuracy of 89.30%, surpassing SVM and LSTM by approximately 13.44%. Meanwhile, as the experimental field size increases, the proposed model demonstrates higher prediction accuracy, reaching a maximum of 98.71%. This study is anticipated to offer early warnings of potential factors affecting crop yields and to further advocate for the adoption of MSIF-based CYP. These findings hold significant research implications for personnel involved in Agricultural and Forestry Economic Management within the context of developing agricultural economy.

## Introduction

Globally, the enduring challenge of food security, particularly in regions with limited food resources, such as North Africa and the Middle East, which are heavily reliant on food imports, remains a century-old issue. Even in developed nations, the specter of famine persists. The evolution of time has imbued new dimensions into the concept of food security^1^. Predominantly, food scarcity serves as the primary catalyst for famine, triggering a cascade of issues such as the escalation of global food prices. Notably, geopolitical conflicts, trade restrictions, economic sanctions, and a decelerating global economy can lead to significant spikes, with wheat and flour prices in North Africa soaring by over 100% and corn prices escalating by more than 70%^2,3^. In 2020, the Food and Agriculture Organization (FAO) of the United Nations (UN) and the World Food Programme (WFP) jointly published an early warning analysis report on regions experiencing extreme food insecurity^4^. The report underscores that without intervention, populations in Burkina Faso, northeastern Nigeria, southern Sudan, and parts of Yemen may imminently face famine if the situation continues to deteriorate^5^. Escalation of conflicts or impediments to humanitarian assistance could elevate the risk of famine. On a global scale, regions facing food insecurity have expanded from the initial four countries to 16, all at a high risk of extreme hunger^6^.

In 2022, the WFP appealed for donations to address the global famine crisis. However, recognizing the intricate nature of global famine factors, financial support alone can only alleviate surface-level problems without eradicating them^7^. As outlined in the 2022–2023 Global Agricultural Product Supply and Demand Forecast Report, global corn and soybean yields have diminished since February 2022, while global wheat and rice yields have increased. The structural changes can be attributed to ongoing declines in corn and soybean production in Argentina due to drought, wheat production in Russia and Ukraine, and increased rice production. Specifically, corn yield, exports, and consumption have all experienced reductions while wheat production continues to rise. Month on Month (MoM), soybean production, crushing capacity, and inventory have decreased, while rice production and consumption have increased^8^. The report also underscores risks such as natural disasters, climate change, geopolitical tensions, and import–export policies. Ukraine, heavily reliant on corn as its primary export, faces a potential decrease in corn yield from 25.6 million tons in 2022 to 21.7 million tons in 2023, with the expected reduction in sown area from over 4 million hectares to 3.6 million hectares due to the Russia Ukraine conflict^9^.

This study aims to scientifically guide the problem of food distribution by timely and accurate allocation of corn resources, and effectively improve the accuracy and application stability of corn yield prediction. The study focuses on experimental corn fields of different sizes, including plots of 1, 10, 20, 30, and 50 hectares. These fields of different sizes are selected to comprehensively evaluate the adaptability and predictive power of the model at various scales. This helps to better understand the performance of the model in diverse spatial dimensions and provides more comprehensive research results for the management of agricultural fields and agricultural economic systems at different scales. The results could offer early warning of factors that may affect crop yields and further promote the use of multivariate information data to predict corn yields. It has important research significance for the Agricultural & Forestry Economic Management (AFEM) and the development and construction of the agricultural economy. This study looks at the immediate causes of food shortages and delves into how these challenges can be addressed by improving crop yield prediction (CYP) models to ensure the sustainability of agricultural production.

In summary, the first paragraph of the introduction describes the challenges to global food security, particularly how food shortages can be a catalyst for famine and rising global food prices. The second paragraph elaborates on the multiple factors leading to food shortages, such as climate change, geopolitical tensions, etc., which exacerbate food insecurity. The third paragraph indicates that the CYP model must be improved to address these challenges effectively. This study aims to develop a more accurate prediction model for corn yields to better manage resources and afford early warning to mitigate the impact of food shortages. These three paragraphs are closely linked and together form this study's background, questions, and objectives.

## Literature review

### Machine learning-based CYP

In research on the application of machine learning methods in different fields, Zhang et al.^10^ amalgamated the deep belief network (DBN) and support vector machine (SVM) for cyberattack detection, achieving a notable level of accuracy. In a separate study, Volpato et al.^11^ employed the Kalman filter to estimate the winter wheat yield at a national level, employing the Normalized Difference Vegetation Index (NDVI) to standardize the time series model. Additionally, Beguería et al.^12^ utilized a gray model based on arable land data in Jilin Province, China, for predicting grain yield. The model considered key factors influencing grain production, including fertilizer usage, livestock, and acreage, resulting in a partial Mean Absolute Percentage Error (MAPE) of 6.67% and an overall MAPE of 5.20% within Jilin Province^12^. Kross et al.^13^ predicted the relative yield of summer crops harvested in 2018 using remote sensors and multiple regression models. Their findings revealed that 20% of CYP errors were below 2%, and 40% were less than 5%^13^. Similarly, Olson et al.^14^ applied remote sensor-based CYP under the Crop Water Stress (CWS) scale, indicating effective CWS and soil moisture using the temperature vegetation drought index. Their study explored the interplay between temperature vegetation dryness index, solar radiation, and yields of winter crops in humid areas. Results demonstrated a Mean Relative Error (MRE) of 13.34%, with incident radiation significantly impacting crops in such regions^14^. Lin et al.^15^ proposed an SVM-enabled World Food Studies (WOFOST) grain yield prediction model, using corn yield in Changchun City, China, as an illustrative example. Comparative testing against independent SVM models revealed the proposed model’s superior accuracy, particularly in predicting crop disasters^15^. Furthermore, PS et al.^16^ integrated Principal Component Analysis (PCA) and Extreme Learning Machine (ELM) to predict short-term grain yields. Their comparison of predicted results with actual data yielded an MRE of 1.90% and 2.08% for short-term grain yield over three and five years, respectively. Simultaneously, accurate short-term grain yield predictions were achieved using the Backpropagation Neural Network (BPNN)^16^.

### CYP using multi-source information fusion

In the realm of multi-source information fusion (MSIF)-based CYP research, Shook et al.^17^ utilized Remote Sensing Technology (RST) and satellites to monitor winter wheat growth. Their work introduced a winter wheat remote monitoring system and a yield prediction system strategically designed to safeguard the interests of farmers^17^. Building on extensive experimentation, Murtaza et al.^18^ integrated RST and Geographic Information System (GIS) to develop a comprehensive CYP system. Ji et al.^19^ integrated artificial neural networks (ANNs) and statistical methods into CYP models, incorporating various vegetation coefficients. Sharifi et al.^20^ completed a regression analysis for US crop yield based on the NDVI, normalized water index, and dual-band enhanced vegetation index, achieving highly accurate results. Wolanin et al.^21^ formulated a corn yield regression equation by combining process model theory and RST during their study of corn yield in the Northeast agricultural lands of China. Their successful predictions aided local farmers in planning effective strategies^21^. Nevavuori et al.^22^ predicted winter wheat yield by linear regression models, incorporating resampling particle filter algorithms with county-level univariate data. They identified influencing factors related to specific management models affecting winter wheat yield per unit area^22^. Abdel Fattah et al.^23^ utilized multi-temporal Unmanned Aerial Vehicle (UAV) remote sensing data to predict summer corn yield, demonstrating the superior predictive efficacy of multi-generational remote sensing over single-generation long-term predictions. Hara et al.^24^ leveraged meteorological data to analyze soil water content, employing multi-linear regression to derive an optimal model. The resulting simple equation, characterized by coefficients that aptly explained and accurately estimated crop yield, showcased promising results^24^. Archontoulis et al.^25^ delved into the climate impact on seasonal CYP, incorporating dynamic factors like temperature, radiation, and rainfall. Their study revealed the significant influence of the proposed dynamic climate model on crop yield^25^. Meanwhile, Dang et al.^26^ established a rice yield regression model utilizing a Random Forest algorithm, primarily considering the rice spectral index. Although the proposed model demonstrated simplicity, ease of data acquisition, and high implementation efficiency, its limited robustness and failure to consider other characteristics of crop yield formation posed challenges in interpreting and analyzing yield prediction results^26^.

Obviously, scholars have amalgamated MSIF and machine learning methodologies for CYP research, predominantly acquiring multi-source data through hyperspectral images, drone images, etc. However, operational constraints and elevated costs persist in practical applications. In the context of CYP research, the deployment of high-resolution cameras emerges as a viable alternative for crop monitoring. Nonetheless, utilizing a singular machine learning approach for CYP emphasizes internal influencing factors of crops while overlooking external factors. The term “internal influencing factors” refers to intrinsic elements that affect crop growth and yield, such as soil quality, moisture levels, and fertilization. On the other hand, “external influencing factors” pertain to the impact of environmental elements on crop growth and yield, including climate, weather conditions, and pest infestations. This study focuses on how to simultaneously consider and analyze these internal and external factors to conduct a more comprehensive prediction of crop yield. Moreover, neural network models are susceptible to data limitations and may not comprehensively encapsulate the myriad factors influencing grain production, leading to substantial prediction errors. Based on the above analysis, this study endeavors to address the prevailing deficiencies in the existing literature, provide insights into the factors impacting crop yield (specifically corn), and advocate for the broader adoption of the MSIF technique in CYP. In order to achieve this objective, the Random Forest methodology is introduced to mitigate model overfitting and enhance noise robustness. The uniqueness of this study in employing Random Forest lies in its application to address model overfitting and enhance noise robustness. Specifically, the introduction of the MSIF technique in the Random Forest model combines with a volatile corn yield dataset, allowing the model to consider the influence of various internal and external factors on crop yield. Consequently, in this study, Random Forest is not merely utilized as a tool but is integrated with the MSIF technique to improve the accuracy and reliability of the model. Overall, this study anticipates corn yield within a designated geographical area with meticulous consideration for spatial variability. The research critically examines the fitting degree and CYP capabilities of the Random Forest model. Empirical findings indicate a notably high level of prediction accuracy and commendable reliability.

## Research methodology

### The core idea of the Random Forest algorithm

**Figure 1:**
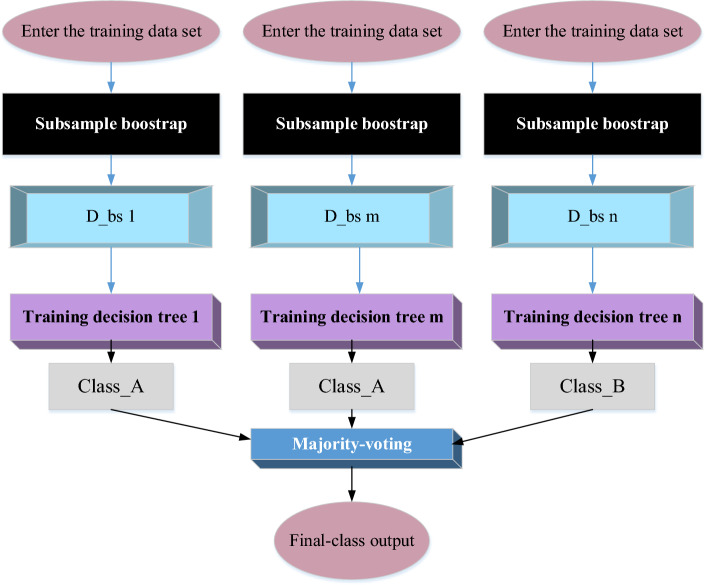
The Random Forest training flow (Drawing software: Visio 2013).

The Random Forest constitutes an amalgamated machine learning algorithm rooted in the aggregation of output from multiple decision trees to yield an enhanced outcome. Distinctively refining the “bagging” technique, the Random Forest assembles a robust learner through the simultaneous deployment of numerous parallel yet independent identical weak learners. In classification tasks, the cumulative votes of individual weak classifiers collectively determine the result. In contrast, for regression problems, the Random Forest algorithm computes the mean of the output from weak learners, addressing the inherent characteristics of CYP as a representative regression problem. Hence, this research opts for the Random Forest methodology, employing “bagging” across multiple binary decision trees^27,28^. The training process of a Random Forest is depicted in Fig. 1.

**Figure 2:**
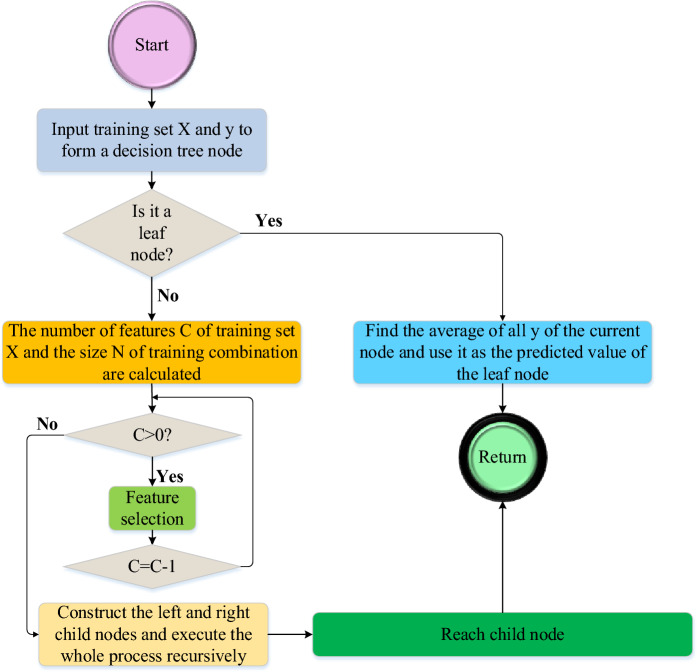
The decision tree’s training flow (Drawing software: Visio 2013).

In Fig. 1, a Random Forest amalgamates the outputs of a collection of independent decision trees by posing a sequence of yes/no queries about elements within the dataset, culminating in the final result. The prediction probability is directly proportional to the quantity of decision trees integrated into the Random Forest model. Given that a Random Forest encapsulates the collective decisions of the majority of its constituent trees, the resultant outcome surpasses that of any individual member. Meanwhile, the voting process among member trees safeguards against potential harm, as it curtails errors and prevents adverse interactions between individual trees. Figure 2 elucidates the specific training process of an individual decision tree.

In Fig. 2, the training of the decision tree involves the careful consideration of feature and segmentation point selection and evaluation. In this context, a comprehensive testing method is employed for the identification of features and segmentation points. Specifically, the procedure involves traversing all values of the *C*-th feature within the training set. Each value serves as a segmentation point, and its efficacy post-segmentation is computed. Subsequently, the segmentation complexity of each point is compared with the minimum complexity of the current node. If the former is found to be smaller than the latter, the segmentation points and corresponding segmentation features are stored. Following the determination of the optimal segmentation, the training set is bifurcated into two sets: the left subnode and the right subnode. The entire segmentation process is iteratively executed until all sub-nodes are reached and returned^29,30^. The purity of the segmented nodes, as measured by Eq. (1), gauges the quality of features and segmentation points:

1 $$ G\left( {x_{i} ,v_{ij} } \right) = \frac{{n_{{{\text{left}}}} }}{{N_{s} }}H\left( {X_{{{\text{left}}}} } \right) + \frac{{n_{{{\text{right}}}} }}{{N_{s} }}H\left( {X_{{{\text{right}}}} } \right) $$

In Eq. (1), *x*_*i*_ and *v*_*ij*_ represent the segmented variable and its respective segmented value, respectively. *n*_*left*_ and *n*_*right*_ denote the number of left and right sub-nodes of the training samples. *N*_*s*_ signifies the total number of sub-nodes within the training sample. The function *H*(*X*) denotes the node impurity function. Equations (2) and (3) illustrate two frequently employed impurity functions tailored for regression problems.

2 $$ H\left( {X_{m} } \right) = \frac{1}{{N_{m} }}\mathop \sum \limits_{{i \in N_{m} }} \left( {y - \overline{{y_{m} }} } \right)^{2} $$

3 $$ H\left( {X_{m} } \right) = \frac{1}{{N_{m} }}\mathop \sum \limits_{{i \in N_{m} }} \left( {y - \overline{{y_{m} }} } \right) $$

Equations (2) and (3) compute the Mean Square Error (MSE) and the Mean Absolute Error (MAE), respectively. *N*_*m*_ corresponds the number of nodes in the training sample. *y* and $\overline{{y_{m} }}$ represent the true value and the predicted value for regression, respectively. The prediction of corn yield in this research, the MSE study is chosen. Equation (4) illustrates the regression results for a specific segmentation point:

4 $$ G\left( {x,v} \right) = \frac{1}{{N_{s} }}\left( {\mathop \sum \limits_{{y_{i} \in X_{left} }} \left( {y_{i} - \overline{{y_{{{\text{left}}}} }} } \right)^{2} + \mathop \sum \limits_{{y_{j} \in X_{right} }} \left( {y_{j} - \overline{{y_{{{\text{right}}}} }} } \right)^{2} } \right) $$

In Eq. (4), *G*(*x*, *v*) represents the weighted sum of the impurity levels across each node. *N*_*s*_ denotes the number of sub-nodes in the training samples. The variables y_*i*_ and *y*_*j*_ denote the actual value of nodes *i* and *j*, respectively. Additionally, $\overline{{y_{left} }}$ and $\overline{{y_{right} }}$ represent the summation of training samples for dividing the left node *i* and the right node *j*. Following the establishment of decision trees, the classification outcome of Random Forest is computed using Eq. (5):

5 $$ H\left( x \right) = \mathop {\mathop {argmax}\limits_{Y} }\limits_{{}} \mathop \sum \limits^{{\mathop {i = 1}\limits_{k} }} W\left( {h_{i} \left( x \right) = Y} \right) $$

In Eq. (5), *H*(*x*) signifies the ultimate outcome derived from the Random Forest. *W* represents the Classification and Regression Tree (CART) model. The term *h*_*i*_(*x*) denotes the classification model for each individual decision tree, and *Y* represents the classification result of *h*_*i*_(*x*).

Random Forests demonstrate proficiency in managing high-dimensional data, where the significance of trained features plays a pivotal role in influencing prediction outcomes^31^. The computation for the importance of a node *k* in a Random Forest is expressed as shown in Eq. (6):

6 $$ n_{k} = w_{k} G_{k} - w_{{{\text{left}}}} G_{{{\text{left}}}} - w_{{{\text{right}}}} G_{{{\text{right}}}} $$

In Eq. (6), *w*_*k*_ represents the ratio of the number of training samples at node *k* to the total number of training samples. Likewise, *w*_*left*_ and *w*_*right*_ denote the ratios of the number of training samples on the left subnode and the right subnode to the total number of training samples, respectively. Additionally, *G*_*k*_, *G*_*left*_, and *G*_*right*_ signify the impurity levels of node *k*, the left subnode, and the right subnode, respectively. The computation for feature importance is articulated as shown in Eq. (7):

7 $$ f_{i} = \frac{{\sum\nolimits_{{j \in feature\,i\,{\text{tangent}}\,{\text{point}}\,n_{j} }} {} }}{{\sum\nolimits_{{k \in {\text{ all}}\,{\text{nodes}}}} {n_{k} } }} $$

In Eq. (7), *n*_*k*_ denotes the collective importance of all nodes, while *n*_*j*_ corresponds to the point $\left( {i{ \ni }j} \right)$ where feature *i* is segmented. Ultimately, the importance of features undergoes normalization, ensuring that their cumulative sum equates to 1. The precise calculation is elucidated as shown in Eq. (8):

8 $$ f_{ni} = \frac{{f_{i} }}{{\mathop \sum \limits_{{j \in {\text{ total features}}}} f_{j} }} $$

### MSIF-based corn yield data collection

**Figure 3:**
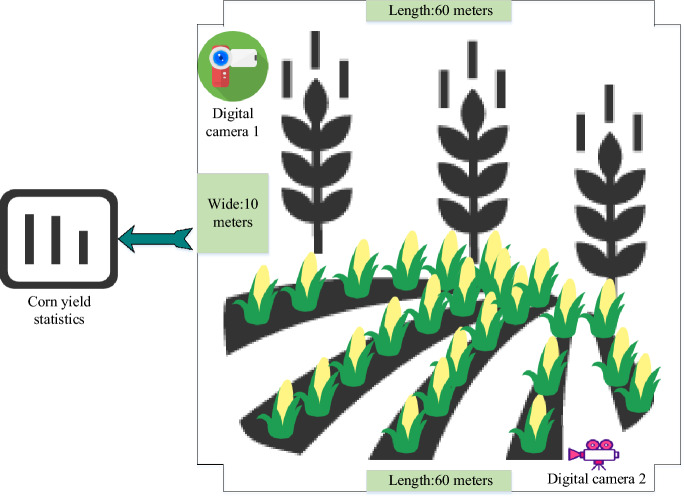
Digital images of the cornfield captured by the camera (Drawing software: Visio 2013).

In order to prognosticate corn yield, this section deploys a digital camera positioned above the experimental field, capturing a comprehensive image of the cornfield measuring 60 m in length and 10 m in width. Subsequently, data pertaining to corn yield, development, and growth within the specified area are incorporated as a source of multi-source information for CYP. Figure 3 illustrates the digital image of the experimental cornfield.

**Figure 4:**
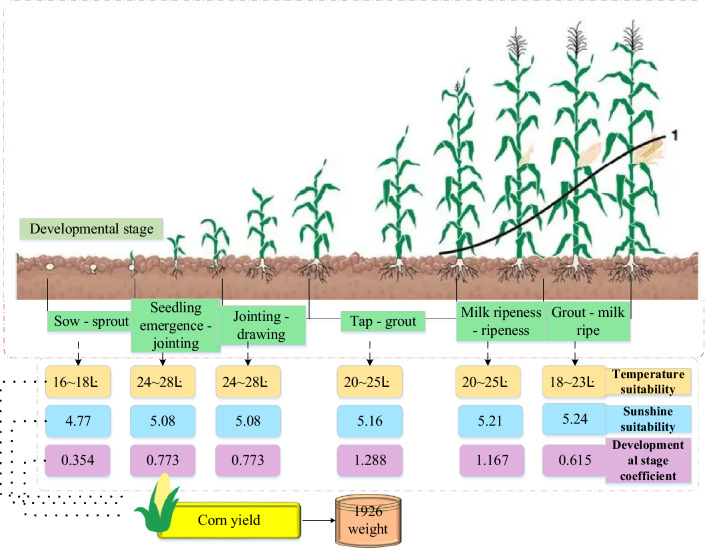
Multi-source information on corn yield in the study area (Drawing software: Visio 2013).

In Fig. 3, datasets from the cornfield’s RGB (Red, Green, and Blue) and hyperspectral camera comprise Portable Network Graphics (PNG ) and Matrix (MAT ) file formats. The analysis of multi-source information from the experimental cornfield is depicted in Fig. 4.

**Figure 5:**
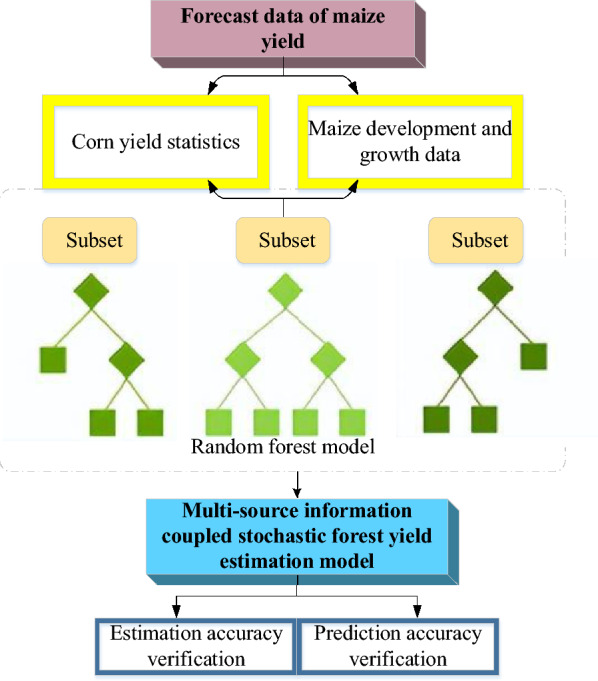
An MSIF-based CYP Random Forest model (Drawing software: Visio 2013).

Within Fig. 4, the growth and development of corn are categorized into six stages. Notably, optimal conditions for corn growth are observed in the sowing and germination stage, characterized by a temperature range of 16 to 18 °C, sunshine suitability of 4.77, and a crop coefficient of 0.354. The germination and jointing stage and jointing and tasseling stage exhibit optimal conditions with a growth temperature of 24–28 °C, sunshine suitability of 5.08, and a crop coefficient of 0.773. In the tasseling and filling, the corn thrives under a suitable temperature of 20–25 °C, sunshine suitability of 5.16, and a crop coefficient of 1.288. Similarly, the filling and milk stages benefit from a suitable growth temperature, sunshine suitability, and crop coefficient of 20 to 25 °C, 5.21, and 1.167, respectively. Lastly, the milk and mature stage demonstrates optimal growth conditions at a temperature range of 18 to 23 °C, sunshine suitability of 5.24, and a crop coefficient of 0.615. Referring to El-Hendawy et al. ’s^32^ study, spring wheat yield can reach up to 1050 Jin under suitable conditions encompassing soil fertility, climate, vegetation variety, and field management. Spring wheat yield and land productivity under different conditions were estimated by a multivariate ensemble model integrating biophysical parameters and hyperspectral index^32^. Therefore, based on pertinent data, an estimate suggests that the 30 m × 10 m crop field could yield between 1400 and 2200 Jin. In alignment with the experimental cornfield in this study, the corn yield also falls within the range of this study 1400–2200 Jin. Consequently, the implementation of digital image-based CYP is considered reasonable. Based on this premise, a CYP Random Forest model is executed using the corn fluctuation yield dataset. Figure 5 illustrates the proposed MSIF-based CYP Random Forest model.

### Experimental preparation

The experimental environment is configured with the Windows 10 Operating System, featuring the AMD R7-5800H 3.2 GHz Central Processing Unit (CPU ), 16 GB Random Access Memory (RAM ), Python 3.6, and a development integration environment of Python 1.3. The camera utilized in the experiment is a high-altitude parabolic network camera with a resolution of 2560 × 1440, equipped with a 1/1.8-inch black light level image sensor and an F1.4 large aperture lens. This camera supports parabolic event alarm, trajectory rendering, message push, video viewing, intelligent perimeter defense, and motion detection. Furthermore, for assessing the performance of the proposed MSIF-based CYP Random Forest model, evaluation metrics such as MSE, Root Mean Squared Error (RMSE ), and determinant coefficient *R*^2^ are selected as indicators. The specific calculation for RMSE and *R*^2^ are articulated as shown in Eqs. (9) and (10):

9 $$ R^{2} = 1 - \frac{{\mathop \sum \limits^{{\mathop {i = 1}\limits_{n} }} \left( {y_{i} - \widehat{{y_{l} }}} \right)^{2} }}{{\mathop \sum \limits^{{\mathop {i = 1}\limits_{n} }} \left( {y_{i} - \overline{{y_{l} }} } \right)^{2} }} $$

10 $$ RMSE = \sqrt {\frac{{\mathop \sum \limits^{{\mathop {i = 1}\limits_{n} }} \left( {y_{i} - \widehat{{y_{l} }}} \right)^{2} }}{n}} $$

In Eqs. (9) and (10), *n* represents the number of samples, with the *i*th sample denoted by *i*, and *y*_*i*_ representing the actual production data for the *i*th sample. The simulated yield of the ith sample is denoted by $\widehat{{y_{i} }}$, while *y*_*i*_ signifies the average of the sample data. The evaluation indexes for assessing the model’s performance are calculated through Eqs. (11) and (12):

11 $$ AE = \left| {y_{p} - y_{r} } \right| $$

12 $$ {\text{Accuracy }} = 1 - \left| {\frac{{y_{p} - y_{r} }}{{y_{r} }}} \right| $$

In Eqs. (11)-(12), *AE*, *y*_*r*_, and *y*_*p*_ represents the absolute error, the actual output, and the simulated output.

## Results and discussions

### Performance analysis of an MSIF-based CYP Random Forest model

**Figure 6:**
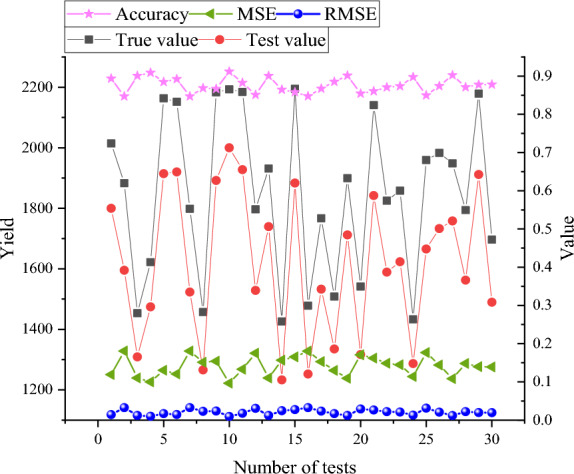
Performance results of the proposed MSIF-based CYP Random Forest Model (Drawing software: Origin 2021).

This section utilizes the data from the 60 m × 10 m experimental field data as input for the prediction of corn yield. The performance results are illustrated in Fig. 6.

**Figure 7:**
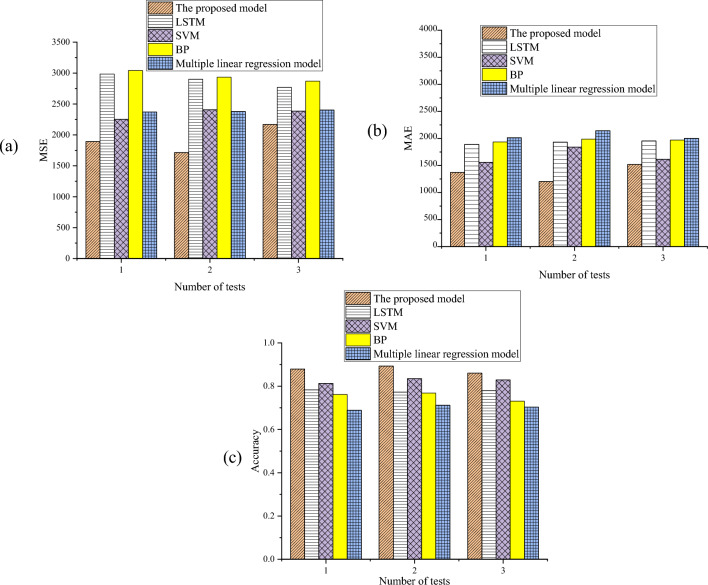
The performance comparison of the proposed MSIF-based CYP Random Forest and control models [(**a**) MSE; (**b**) the MAE value; and (**c**) *Accuracy*] (Drawing software: Origin 2021).

As depicted in Fig. 6, the corn yield within the experimental area spans from 1400 to 2200 Jin, whereas the predicted corn yield varies between 1350 to 2100 Jin. Notably, the average yield for the experimental area is 1820.72 Jin, contrasting with the predicted corn yield of 1602.765 Jin. The average accuracy of the proposed MSIF-based CYP Random Forest model is 87.72%. Moreover, the model’s MSE and RMSE are computed as 0.14 and 0.0196, respectively. Furthermore, SVM, Long Short-Term Memory (LSTM), BPNN, and Multiple Linear Regression (MLR) are designated as control models. A comparative analysis of the performance between the control models and the proposed CYP Random Forest model is presented in Fig. 7.

According to Fig. 7, the proposed CYP Random Forest model exhibits the smallest MSE and MAE on the same test sample, surpassing the BPNN model with the largest error. The error magnitude of the proposed model is lesser compared to the control models, indicating a better reflection of the actual situation. Moreover, as illustrated in Fig. 7c, the highest prediction accuracy achieved by the proposed CYP model is 89.30%, outperforming LSTM, SVM, BPNN, and MLR with prediction accuracies of 78.30%, 83.50%, 76.80%, and 71.20%, respectively. In summary, the proposed MSIF-based CYP Random Forest model demonstrates superior performance, surpassing SVM and LSTM by a prediction accuracy margin of 13.44%.

### Validation of the proposed MSIF-based CYP Random Forest model

**Figure 8:**
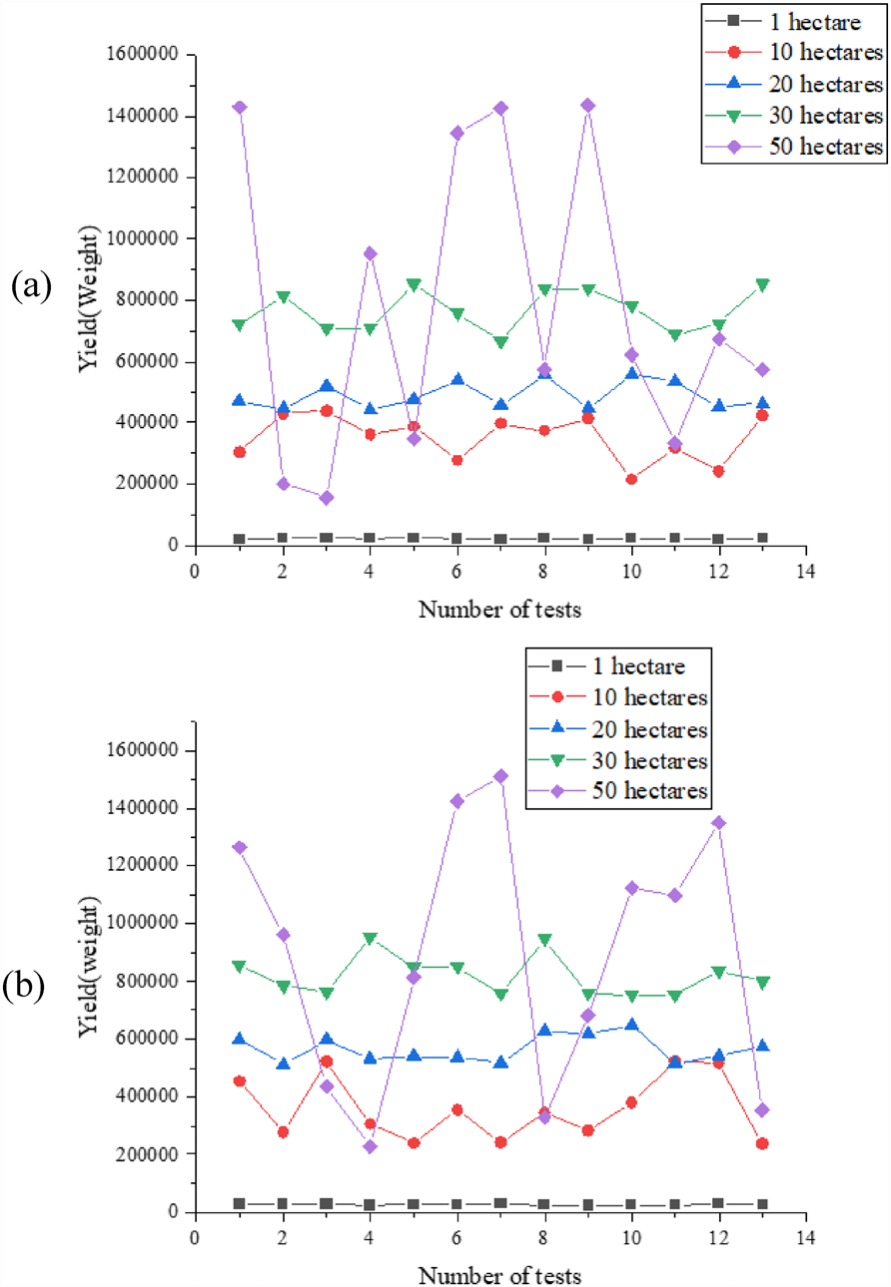
The CYP results of different-sized experimental fields [(**a**) the predicted values; (**b**) the true value] (Drawing software: Origin 2021).

Subsequently, the fitting degree and prediction ability of the proposed MSIF-based CYP Random Forest model are scrutinized using statistical corn yield data as a verification dataset. Specifically, the corn yield is predicted within the 1-hectare, 10-hectare, 20-hectare, 30-hectare, and 50-hectare experimental fields, and the results are illustrated in Fig. 8.

**Figure 9:**
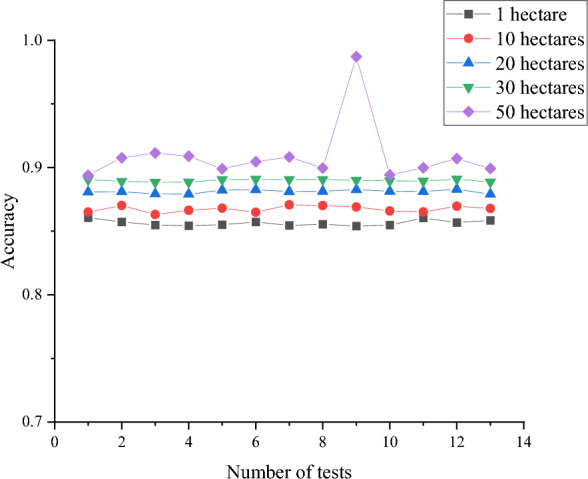
Accuracy of the proposed MSIF-based CYP Random Forest model on different-sized experimental fields (Drawing software: Origin 2021).

Figure 8 illustrates that the CYP curve for various areas aligns closely with the true value. Specifically, on a 1-hectare, 10-hectare, 20-hectare, 30-hectare, and 50-hectare fields, the predicted corn yield ranges from 19,680.4–25,814.92 Jin, 217,263.7–438,867.9 Jin, 443,898.6–559,433.2 Jin, 668,475.7–853,015.6 Jin to 157,907.1–1,436,498 Jin. The forecasted corn yield for different-sized fields falls within the actual value range. The accuracy of the proposed MSIF-based CYP Random Forest model is depicted in Fig. 9.

According to Fig. 9, the accuracy of the proposed MSIF-based CYP Random Forest model on 1-hectare, 10-hectare, 20-hectare, 30-hectare, and 50-hectare fields is 85.81%, 86.57%, 88.12%, 88.98%, and 90.35%, respectively. Moreover, as the experimental field expands in size, the proposed MSIF-based CYP Random Forest model demonstrates higher accuracy, reaching a maximum of 98.71%

## Conclusion

In order to advance the application of the MSIF technique in CYP and agricultural and forestry management, this study introduces the Random Forest method to predict corn yield, taking into account its spatial variation. Consequently, the MSIF-based CYP Random Forest model is proposed, and its fitting degree and prediction ability are evaluated, yielding highly accurate prediction results. The research findings reveal that the proposed model achieves a peak prediction accuracy of 89.30%. Specifically, the accuracy on 1-hectare, 10-hectare, 20-hectare, 30-hectare, and 50-hectare test fields reaches 85.81%, 86.57%, 88.12%, 88.98%, and 90.35%, respectively. Therefore, the proposed MSIF-based CYP Random Forest model proves effective in predicting corn yield. Finally, it is essential to acknowledge certain research limitations, such as the omission of regional and terrain differences and various other factors influencing corn yield. Future research endeavors should aim to incorporate additional factors for a more accurate prediction of corn yield in the study (Supplementary Informations 1, 2, 3, 4).

### Supplementary Information

Supplementary Information 1.

Supplementary Information 2.

Supplementary Information 3.

Supplementary Information 4.

## Data Availability

All data generated or analyzed during this study are included in this published article [and its supplementary information files].
