# Supplementary material for: Multi-source information fusion-driven corn yield prediction using the Random Forest from the perspective of Agricultural and Forestry Economic Management
Source: Sci Rep. 2024 Feb 19;14:4052. doi: 10.1038/s41598-024-54354-9 (PMC11325042; doi:10.1038/s41598-024-54354-9)
Supplement: Supplementary file 1 — Supplementary Information 1. [file 41598_2024_54354_MOESM1_ESM.docx]

Number of tests True value Test value Accuracy MSE RMSE

1 2014.16325 1799.81795 0.89358 0.11909 0.01418

2 1883.12027 1594.96461 0.84698 0.18067 0.03264

3 1453.00683 1308.81974 0.90077 0.11017 0.01214

4 1621.9254 1474.30104 0.90898 0.10013 0.01003

5 2163.66665 1914.1498 0.88468 0.13035 0.01699

6 2151.6883 1919.98109 0.89231 0.12068 0.01456

7 1797.98179 1523.02369 0.84707 0.18053 0.03259

8 1457.25564 1265.82998 0.86864 0.15123 0.02287

9 2183.009 1891.82905 0.86662 0.15391 0.02369

10 2192.92551 1999.75758 0.91191 0.0966 0.00933

11 2184.2954 1927.65048 0.8825 0.13314 0.01773

12 1796.38658 1528.6765 0.85097 0.17513 0.03067

13 1931.23729 1739.35396 0.90064 0.11032 0.01217

14 1426.07203 1232.82603 0.86449 0.15675 0.02457

15 2194.60065 1883.21461 0.85811 0.16535 0.02734

16 1477.89706 1252.19781 0.84728 0.18024 0.03249

17 1766.88041 1532.44041 0.86731 0.15298 0.0234

18 1508.16389 1334.8905 0.88511 0.1298 0.01685

19 1899.41506 1712.02686 0.90134 0.10945 0.01198

20 1541.4191 1316.80111 0.85428 0.17058 0.0291

21 2140.6484 1841.86263 0.86042 0.16222 0.02632

22 1825.01445 1588.9069 0.87063 0.1486 0.02208

23 1858.28262 1623.37443 0.87359 0.1447 0.02094

24 1433.24 1286.64079 0.89771 0.11394 0.01298

25 1959.57773 1665.30746 0.84983 0.17671 0.03123

26 1982.68466 1732.88402 0.87401 0.14415 0.02078

27 1948.31814 1758.01019 0.90232 0.10825 0.01172

28 1793.94808 1562.32963 0.87089 0.14825 0.02198

29 2178.79885 1911.43448 0.87729 0.13988 0.01957

30 1696.4154 1489.45311 0.878 0.13895 0.01931
