# Supplementary material for: Multi-source information fusion-driven corn yield prediction using the Random Forest from the perspective of Agricultural and Forestry Economic Management
Source: Sci Rep. 2024 Feb 19;14:4052. doi: 10.1038/s41598-024-54354-9 (PMC11325042; doi:10.1038/s41598-024-54354-9)
Supplement: Supplementary file 2 — Supplementary Information 2. [file 41598_2024_54354_MOESM2_ESM.docx]

(a)

Number of tests The proposed model LSTM SVM BP Multiple linear regression model

1 1892.711 2984.583 2252.193 3042.15 2369.41

2 1714.633 2899.374 2407.448 2933.74 2378.66

3 2168.958 2768.356 2385.538 2869.58 2400.98

(b)

Number of tests The proposed model LSTM SVM BP Multiple linear regression model

1 1372.641 1890.358 1559.490 1934.56 2012.23

2 1203.842 1932.583 1841.385 1987.12 2143.58

3 1519.5 1953.683 1613.842 1969.55 2000.87

(c)

Number of tests The proposed model LSTM SVM BP Multiple linear regression model

1 0.879 0.783 0.813 0.762 0.689

2 0.893 0.773 0.835 0.768 0.712

3 0.86 0.779 0.829 0.731 0.703
