# Supplementary material for: Multi-source information fusion-driven corn yield prediction using the Random Forest from the perspective of Agricultural and Forestry Economic Management
Source: Sci Rep. 2024 Feb 19;14:4052. doi: 10.1038/s41598-024-54354-9 (PMC11325042; doi:10.1038/s41598-024-54354-9)
Supplement: Supplementary file 3 — Supplementary Information 3. [file 41598_2024_54354_MOESM3_ESM.docx]

(a)

Number of tests 1 hectare 10 hectares 20 hectares 30 hectares 50 hectares

1 22381.27209 304036.6476 469280.2194 722424.4054 1429779.853

2 25511.79046 431659.6809 446499.1516 816573.649 202335.6628

3 25814.91928 438867.8787 519723.2407 708140.5208 157907.1087

4 24300.15846 362049.1162 443898.6066 708857.1778 953977.3601

5 25721.18521 388957.0859 475286.0534 853015.5945 347281.1826

6 22859.33257 278698.6115 539128.2065 757571.773 1347040.366

7 19949.03995 398008.5316 459683.3561 668475.672 1428291.607

8 24876.99871 374019.9638 558132.6484 836927.9112 574110.5627

9 20018.11509 415974.9393 446905.3355 838237.5477 1436498

10 24654.25804 217263.7492 559433.2277 779947.7225 621701.8996

11 24633.51267 318450.9739 534605.7359 688818.6611 333759.5495

12 19680.39774 244219.9615 450743.4781 725813.5295 675541.7144

13 25787.30531 425254.0997 464094.107 852196.2827 573700.9711

(b)

Number of tests 1 hectare 10 hectares 20 hectares 30 hectares 50 hectares

1 23284.38659 278536.6429 639029.4049 843011.5371 1299929.15

2 24672.66404 541997.9454 553613.628 838132.7739 700285.3637

3 30675.76367 527147.898 614584.5006 767531.4369 982545.0917

4 29963.68877 319235.2927 530218.0078 828301.4729 1006033.969

5 22705.99261 237690.9368 544932.9624 848595.4985 956451.7098

6 22545.05088 396074.9922 585876.1813 812096.6084 561443.0433

7 24300.66776 233470.6234 564069.8626 807189.8352 986315.2479

8 27891.67038 522179.572 565687.5086 797297.5778 136146.8376

9 22965.18672 257058.8833 534728.9191 790960.0477 665378.2715

10 28085.44802 285198.9357 517586.9489 850052.6731 261894.972

11 23508.39208 444876.2156 625369.7358 848531.263 273106.9962

12 31336.78113 387289.2208 575494.5753 904065.4399 660560.2686

13 28575.34538 454590.3402 613169.6436 909013.0864 444937.2327
