# Supplementary material for: Multi-source information fusion-driven corn yield prediction using the Random Forest from the perspective of Agricultural and Forestry Economic Management
Source: Sci Rep. 2024 Feb 19;14:4052. doi: 10.1038/s41598-024-54354-9 (PMC11325042; doi:10.1038/s41598-024-54354-9)
Supplement: Supplementary file 4 — Supplementary Information 4. [file 41598_2024_54354_MOESM4_ESM.docx]

Number of tests 1 hectare 10 hectares 20 hectares 30 hectares 50 hectares

1 0.858567992 0.86369869 0.882347395 0.890244117 0.911353041

2 0.854448592 0.865658673 0.880666238 0.889167213 0.897734398

3 0.859892924 0.871375146 0.882905032 0.888443069 0.899434624

4 0.856679299 0.863091069 0.880197055 0.889652868 0.908299872

5 0.854701561 0.862750998 0.880249578 0.8898023 0.905959709

6 0.856886214 0.865609015 0.880341866 0.889359518 0.894449983

7 0.859312978 0.862495286 0.879839303 0.890485341 0.908113122

8 0.854325432 0.866781032 0.882116147 0.889289626 0.910785295

9 0.854747375 0.867728664 0.880713733 0.89105151 0.9871

10 0.860392811 0.866247294 0.880367608 0.890284475 0.895328241

11 0.856542357 0.866894397 0.881637029 0.890322844 0.893588973

12 0.860248593 0.863330393 0.88128559 0.890835864 0.911030098

13 0.855219317 0.870413537 0.881525195 0.890729513 0.909658954
